# Bookmarking the epigenome by retrovirus integration

**DOI:** 10.1186/1742-4690-8-S2-O16

**Published:** 2011-10-03

**Authors:** Filip Senigl, Miroslav Auxt, Jiri Hejnar

**Affiliations:** 1 Institute of Molecular Genetics, Academy of Sciences of the Czech Republic, Vídèská 1083, CZ-14220 Prague 4, Czech Republic

## 

Integration of retroviruses occurs in most genomic regions with weak but statistical significant preferences for target site sequences. HIV-1 preferentially targets genes, particularly the transcriptionally active ones. Avian sarcoma and leukosis viruses (ASLV) integrate with only slight preference for genes while murine leukemia virus uniquely favors integration in close proximity to upstream or downstream transcription start sites. Genomic features as well as the “epigenetic landscape“ at the site of integration are crucial for the outcome of each integration event, i. e. stable long terminal repeat-driven expression of the provirus or silencing and transcriptional suppression. The current availability of assembled genome sequences with epigenomic characteristics and reliable cloning of retrovirus integration sites enables to study the interplay between transcription signals of the retrovirus and enhancing or suppressive influences of the adjacent cellular DNA.

We studied the integration sites of ASLV-derived vectors integrated into normal or DNA methylation-deficient human cells. Integration sites from cell clones containing single provirus were cloned and their genomic features as well as epigenetic landscape were correlated with expression or silencing of the provirus. Dnmt3b turned out to be the major player in silencing the long terminal repeat-driven transcription. Proviruses integrated into H3K4me3-rich CpG islands associated with promoters of active genes display long-term stability of expression and are resistant to the transcriptional silencing after over expression of Dnmt3b. Stability of expression and resistance to the silencing decreases with the distance from transcription start site. By contrast, proviruses integrated into the intergenic regions tend to the spontaneous transcriptional silencing even in DNA methyltransferase-deficient cells.

